# Using FMRI-guided TMS to modulate social cognition in adults with autism

**DOI:** 10.1192/j.eurpsy.2026.10325

**Published:** 2026-07-31

**Authors:** L. Schilbach

**Affiliations:** 1 LVR-Klinikum Düsseldorf, Düsseldorf; 2 LMU, Munich, Germany

## Abstract

**Abstract:**

This talk will introduce the idea of using personalized approaches to brain stimulation by making using of neuroimaging and will describe a study that used fMRI-guided, neuronavigated sham-controlled TMS to modulate social cognition in adults with and without autism. Results of this study, indeed, demonstrate that fMRI-guided excitatory TMS of right temporo-parietal cortex differentially improves social information processing in the verum-patient group as assessed by a computational probabilistic learning task.

**Disclosure of Interest:**

None Declared

